# Deep Brain Stimulation Surgery for Parkinson Disease Coexisting With Communicating Hydrocephalus: A Case Report

**DOI:** 10.3389/fneur.2018.01011

**Published:** 2018-11-23

**Authors:** Carlos Guevara, Jose de Grazia, Pedro Vazquez, Pablo Baabor, Cristián Garrido, Melissa Martinez, Jaime Fuentes, Fabian Piedimonte, Marcos Baabor

**Affiliations:** ^1^Hospital Clínico, Universidad de Chile, Santiago, Chile; ^2^Fundación CENIT, Universidad de Buenos Aires, Buenos Aires, Argentina

**Keywords:** deep brain stimulation (DBS), parkinson disease, idiopathic normal pressure hydrocephalus (iNPH), ventriculomegaly, globus pallidus internus (GPi), communicating hydrocephalus

## Abstract

We report a successful bilateral globus pallidus internus-deep brain stimulation (GPi-DBS) for a Parkinson disease (PD) patient with idiopathic normal pressure hydrocephalus (INPH) and an unusually long anterior commissure-posterior commissure (AC-PC) line. A 54-year-old man presented with a history of 3 months of severe shuffling gait, rigidity, slow movements of the left side limbs, and difficulty managing finances. A brain MRI revealed marked ventriculomegaly (Evans index = 0.42). The patient was diagnosed with INPH and a ventriculoperitoneal shunt was placed. Cognitive impairment improved, but walking disturbances, slowness, and rigidity persisted. Then treatment with levodopa was added, and the patient experienced a sustained improvement. He was diagnosed with PD. After 7 years, the patient developed gait freezing and severe levodopa-induced dyskinesia. The patient underwent bilateral GPi-DBS. We used MRI/CT fusion techniques for anatomical indirect targeting. Indirect targeting is based on standardized stereotactic atlas and on a formula—derived method based on AC-PC landmarks. The AC-PC line was 40 mm (the usual length is between 19 and 32 mm). Intraoperative microelectrode recording was a non-expendable test, but multiple recordings were avoided to reduce the surgical risk of ventricular involvement. There was a 71% decrease in the UPDRS III score during the on-stimulation state (28 to 8). The patient's dyskinesias resolved dramatically with a UdysRS of 15 (88% improvement) during the on-stimulation condition. The observed motor benefits and the improvement of his daily activities have persisted 6 months after surgery. Deep brain stimulation surgery in PD with ventriculomegaly is a challenge. This procedure can result in a greater chance of breaching the ventricle, with risks of intraventricular hemorrhage and migration of cerebrospinal fluid into the brain parenchyma with target displacement. Furthermore, clinical judgment is paramount when recent onset of shuffling gait coexists with ventriculomegaly because the most common dilemma is differentiating between PD and INPH. For these reasons, neurologists and surgeons may refuse to operate on PD patients with ventriculomegaly. However, DBS should be considered for PD patients with motor complications when responsiveness to levodopa is demonstrated, even in the context of marked ventriculomegaly.

## Background

Deep brain stimulation (DBS) surgery in Parkinson's disease (PD) with ventriculomegaly is a challenge. This procedure can result in a greater chance of breaching the ventricle, with risks of intraventricular hemorrhage and migration of cerebrospinal fluid into the brain parenchyma around the leads with subsequent target displacement. Furthermore, clinical judgment is paramount when recent onset of shuffling gait coexists with ventriculomegaly because the most common dilemma is differentiating between PD and idiopathic normal pressure hydrocephalus **(**INPH) (1, 2). For these reasons, neurologists and surgeons may refuse to operate on PD patients with ventriculomegaly. Here, we report a successful bilateral globus pallidus internus-deep brain stimulation (GPi-DBS) for a PD patient with INPH with marked ventriculomegaly and an unusually long anterior and posterior commissure (AC-PC) line.

## Case report

**Figure 1:**
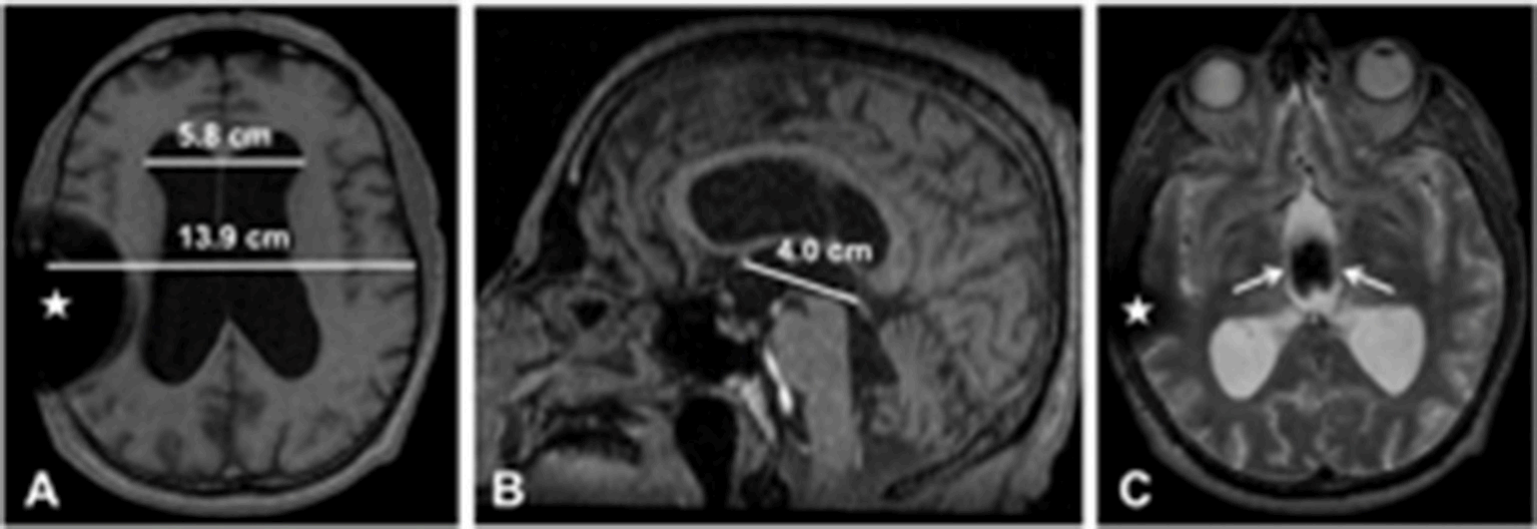
Brain MRI (**A**: T1 axial slice; **B**: T1 sagittal slice; **C**: T2 axial slice). **(A)** There is marked ventriculomegaly. The maximum width of the frontal horns of the lateral ventricles is 5.8 cm; the maximal internal diameter of the skull at the same level is 13.9 cm; the calculated Evans index is 0.42 (normal value: < 0.3). **(B)** The anterior commissure– posterior commissure line is drawn, and its length is 4.0 cm; this line is an important landmark for stereotactic targeting in GPi-DBS. **(C)** The third ventricle is also dilated, and there is prominent flow void artifact (white straight arrows); this artifact means that CSF flow velocity is high and there is no obstruction. Magnetic susceptibility artifacts due to ventriculoperitoneal catheter and valve (white stars).

Fifty-four year-old man presented with a history of 3 months of severe shuffling gait, urinary incontinence, and difficulty managing finances and keeping track of appointments. On examination a soft voice, a delay in left shoulder shrug and mild cogwheel rigidity in the left arm were noticeable. A brain MRI revealed marked ventriculomegaly (calculated Evans index = 0.42) (3) (Figure 1). A lumbar puncture with removal of 50 ml of cerebrospinal fluid (CSF) was performed. The CSF was clear and colorless with an opening pressure of 18 cmH_2_0. After the spinal test, Mini-Mental State Examination scored improved from 23/30 to 29/30 and the patient subjectively experienced significant relief from his gait-related symptoms, showing a 10% of increase in his walking speed. INPH was diagnosed and a ventriculoperitoneal (VP) shunt was placed (Figure 1). Cognitive impairment and urinary incontinence improved, but the parkinsonian features persisted. Then treatment with levodopa was added, and the patient experienced a sustained improvement. It was thought that PD coexisted with INPH. Five years later, he presented with acute headache and cognitive impairment. Shunt obstruction which necessitated surgical intervention was diagnosed. The VP shunt was replaced and these acute symptoms resolved.

After 7 years on levodopa treatment, the patient developed gait freezing and severe levodopa-induced dyskinesia. He was treated with various combinations of trihexyphenidyl, amantadine, pramipexole, and levodopa, up to a maximum tolerated dose (1,500 mg daily), with minimal benefit to his overall functions. At an outside institution, DBS surgery was not considered due to diagnostic and technical concerns; the latter were related to the ventriculomegaly that could complicate the accurate placement of the DBS leads.

**Figure 2:**
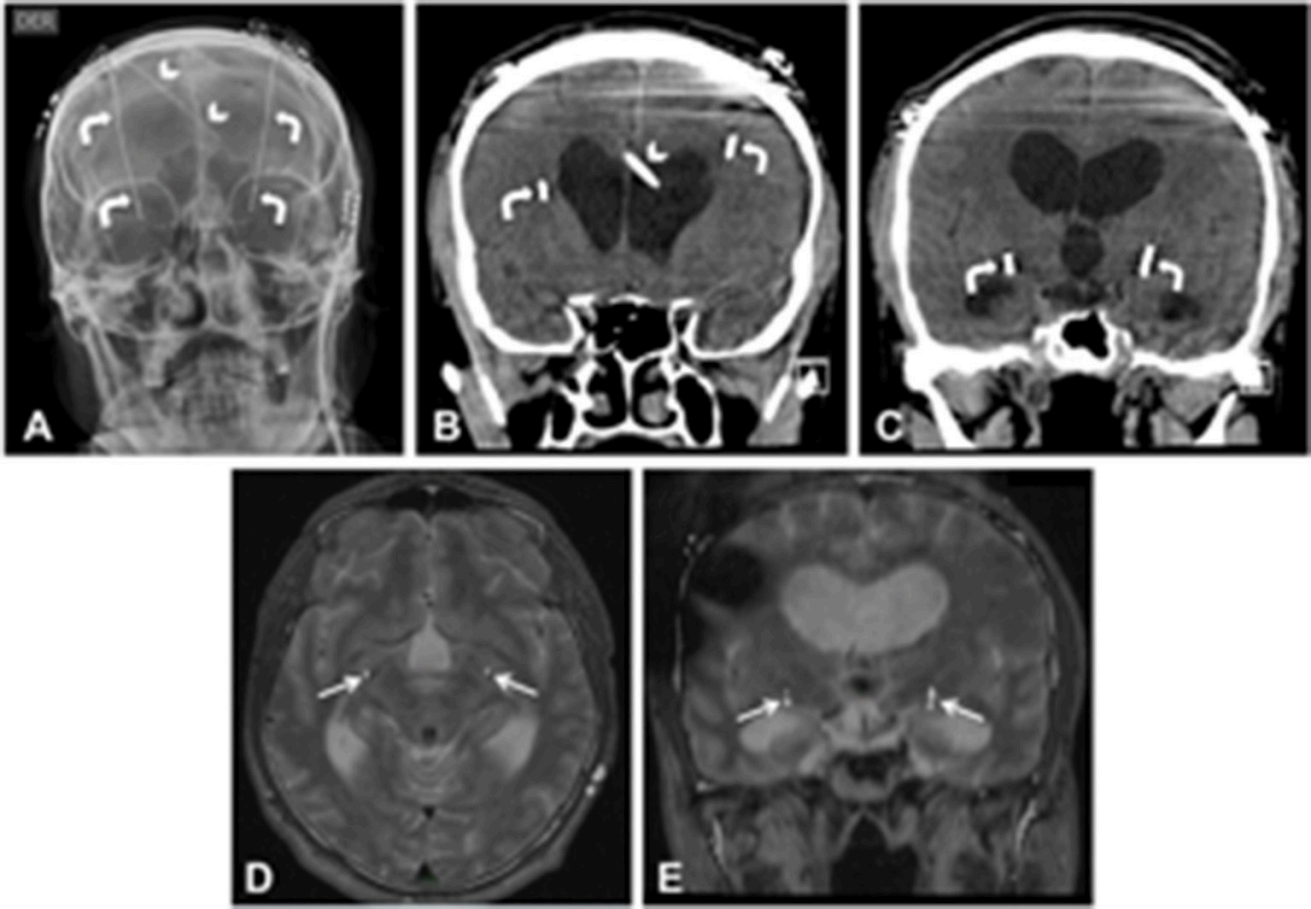
Postoperative GPi-DBS exams (**A**: skull radiography frontal view; **B** and **C**: brain CT coronal slices). The lead tracts (white curved arrows) avoid the right ventriculoperitoneal shunt catheter (white arrowheads) and the enlarged frontal horns of the lateral ventricles. Superimposed fused images between preoperative T2 MRI and postoperative CT (**D**: axial; **E**: coronal) confirmed the lead placement on the GPi bilaterally (white straight arrows).

At the age of 67, the patient was subsequently referred to our institution. Motor score following a levodopa challenge decreased from 45 to 28 on UPDRS III (40% improvement). The Unified Dyskinesia Rating Scale (UDysRS) was very high (128 out of a maximum of 196). A battery of neurocognitive tests showed no signs of cognitive impairment. After a long discussion with the patient and his family on the pro and cons of the surgery, we decided to carry out DBS because the patient was suffering from medically intractable parkinsonism. The patient underwent bilateral GPi-DBS. We chose the ventral GPi in order to suppress dyskinesia and because its more lateral position compared to the subthalamic nucleus reduces the risk of breaching the ventricle walls. We used MRI/CT fusion techniques for anatomical indirect targeting with a stereotactic CT frame. Anterior and posterior commissures were identified, and the AC-PC line was 40 mm (the usual length of the AC-PC line is measured between 19 and 32 mm). The coordinates for the right GPi were 27.38 mm lateral (X), 1.85 mm anterior (Y), and −8 mm inferior (Z) to the midpoint of the intercommisural AC-PC line (10.7° from mid-sagittal plane and 53.3° from axial plane). For the left GPi the coordinates were X: −25.35 mm, Y: 3.69 mm, and Z: −8.42 mm (11.7° from mid-sagittal plane and 67.3° from axial plane). The right burr-hole placement was shifted laterally to avoid the right VP shunt and the enlarged frontal horns of the lateral ventricles (please see Additional File 1: CT video). The number of microelectrode trajectories was reduced to only one. Intraoperative microelectrode recording (MER) identified the neuronal firing pattern of the GPi. Recordings were performed in steps of 0.5–1 mm, from 15 mm above the presumed target until 3 mm below the target. Microstimulation with trains of high-frecuency (200 Hz) and currents from 0.5 to 5 mA was performed in order to test for proximity of the internal capsule, without any noticeable twitching or contraction coincident with stimulation. Visual evoked responses were obtained by a stimulus of light delivered as a flash and the optic tract was identified at the base of both GPi. The GPi-DBS lead placement was confirmed on a postoperative CT scan (Figure 2).

Two weeks after leads placement, monopolar stimulation of the left dorsal and right ventral contacts (1-/11-) at 3.2 V in the left GPi and 3.6 V in the right GPi with a frequency of 130 Hz and pulse duration of 90 ms led to the best clinical response. There was a 71% decrease in the UPDRS III score during the on-stimulation state (28 to 8). The patient's dyskinesias resolved dramatically with a UdysRS of 15 (88% improvement) during the on-stimulation condition. The observed motor benefits and the improvement of his daily activities have persisted 10 months after surgery. (Please see Additional File 2: movie shows the before/after surgery; medication-off/stimulation-on condition).

The subject gave written informed consent for the publication of this case report and video files in accordance with the Declaration of Helsinki.

## Discussion

To our knowledge, this is the first report on DBS in a PD patient with INPH. In the longitudinal care of INPH, the persistent parkinsonism after the shunt surgery and the favorable response to levodopa supported the clinical diagnosis of PD (3). Krauss reported INPH and PD in 4 cases out of 118 adults with hydrocephalus (2). Morishita has emphasized the role of the levodopa challenge for the diagnosis of PD in the setting of ventriculomegaly (1). One may speculate whether this patient had a ventriculomegalic presentation of PD, given the partial response to the VP shunt, the good response to levodopa therapy, and the remarkable response to DBS (4, 5).

We decided to operate on this patient, as DBS was considered the only option to improve his quality of life, although ventriculomegaly that is sufficient to preclude direct electrode passage to the surgical target may be a contraindication to DBS. However, we did not find any absolute contraindication for this surgery, and we found that ventricle volume does not predict motor change following DBS (6). Indirect targeting is based on standardized stereotactic atlas and on a formula—derived method based on AC-PC landmarks. We believed that both indirect targeting and MER would allow us to localize GPi, although the AC-PC line was particularly long, a feature which had never been reported in DBS surgery.

Planning electrode trajectories to avoid sulci and the ventricles was our main precaution. Careful fusion between CT and MRI images and MER to identify the GPi were essential (7). MER was a non-expendable test, but multiple recordings were avoided to reduce the surgical risk of ventricular involvement.

## Concluding remarks

We postulate this case is PD coexisting with INPH. We conclude that DBS should be considered for PD patients with motor complications when responsiveness to levodopa is demonstrated, even in the context of ventriculomegaly.

## Author contributions

CG design of the study, analysis and interpretation of the data, drafting and revising the manuscript for intellectual content. JdG, CG, and JF analysis and interpretation of the data. PV, PB revising the manuscript for intellectual content. MM drafting and revising the manuscript for intellectual content. FP and MB design of the study, analysis and interpretation of the data.

### Conflict of interest statement

The authors declare that the research was conducted in the absence of any commercial or financial relationships that could be construed as a potential conflict of interest.
